# Finding Fusin/CXCR4, the First “2nd Receptor” for HIV Entry

**DOI:** 10.3389/fimmu.2015.00283

**Published:** 2015-06-08

**Authors:** Edward A. Berger

**Affiliations:** ^1^Molecular Structure Section, Laboratory of Viral Diseases, National Institute of Allergy and Infectious Diseases, National Institutes of Health, Bethesda, MD, USA

**Keywords:** coreceptor, tropism, T cell line, macrophage, CCR5, CD4, Env glycoprotein, cell fusion

Shortly after the pioneering Montagnier/Gallo discoveries of HIV as the etiologic agent of AIDS, the CD4 antigen was identified as the primary receptor for HIV entry. The focus of the present story begins with 1986 report from Richard Axel’s group that recombinant human CD4 conferred permissiveness to HIV-1 infection when expressed on diverse human cell types, but not on mouse cells. The block was at an early replication step after virion binding, perhaps virus internalization (1).

My entry into the HIV/AIDS field came at a particularly opportune time (1987) and place (Laboratory of Viral Diseases, NIAID, NIH, headed by Bernie Moss). I was interested in learning the vaccinia virus-based system for recombinant protein expression, and applying it to study HIV entry. Bernie’s group had generated a vaccinia recombinant encoding HIV-1 Env that induced robust CD4-dependent cell fusion as measured by syncytia (2). The strictly cytoplasmic nature of the vaccinia replication cycle turned out to provide a fortuitous advantage for studying Env, since it obviated the as-yet unrecognized need for co-expression of HIV rev to export unspliced Env RNA out of the nucleus; moreover, the extremely broad host range of vaccinia enabled studies of Env-mediated fusion with a variety of cell types from diverse species. In a reductionist system using the corresponding vaccinia recombinants, we showed that cells expressing HIV-1 Env formed syncytia when mixed with cells (lymphoid and non-lymphoid) expressing human CD4, provided the latter were of human origin (3). Parallel results were obtained by other groups (4, 5).

Was this phenomenon due to a requirement for an additional human-specific factor, or to a dominant restrictive feature of the non-human cells? In collaboration with Robert Blumenthal’s group at NCI, NIH, we demonstrated that CD4-expressing transient hybrids between human and murine cells were fusion-permissive, arguing against the non-human restriction model (6). These findings in the reductionist cell fusion system were consistent with studies by others examining HIV infection of transient or stable or cell hybrids (4, 5). Thus, by the early 1990s, it was evident that the CD4-human cell requirement was manifest at the level of Env-CD4-mediated fusion/entry, apparently reflecting target cell expression of an essential human-specific cofactor (perhaps a 2nd receptor, or “coreceptor”).

Further adding to my good fortune was my partnering with postdoc Tom Fuerst in the Moss lab, who had led their development of the vaccinia/T7 hybrid expression system. They had shown that a target gene linked to the phage T7 promoter is activated by the vaccinia-encoded T7 RNA polymerase expressed in the same cell; the presence of all components in the cytoplasm leads to robust transient expression of the target gene (7). I realized that this system could be adapted to study Env-receptor-mediated cell fusion by expressing the vaccinia-encoded T7 polymerase in one cell partner and introducing a reporter gene (e.g., the *E. coli LacZ* gene) linked to the T7 promoter in the other; reporter expression would be triggered in the cytoplasm of fused cells. Postdocs Ofer Nussbaum and Chris Broder in my group demonstrated the highly sensitive and specific nature of this reporter assay and its superiority over the subjective and laborious semi-quantitative syncytium-counting assay (8). Specific fusion was observed when Env-expressing “effector cells” were mixed with CD4-expressing “target cells”; a robust β-galactosidase signal was detected at 2–3 h, either by *in situ* staining or colorimetric assay of detergent cell lysates (Figure 1A). Importantly, the reporter assay corroborated the requirement that CD4 be expressed on a human cell, whereas Env could be on a human or non-human cell. Membrane vesicle transfer experiments demonstrated that the fusion deficiency of CD4-expressing non-human cells was not due to their detrimental modification of CD4.

**Figure 1:**
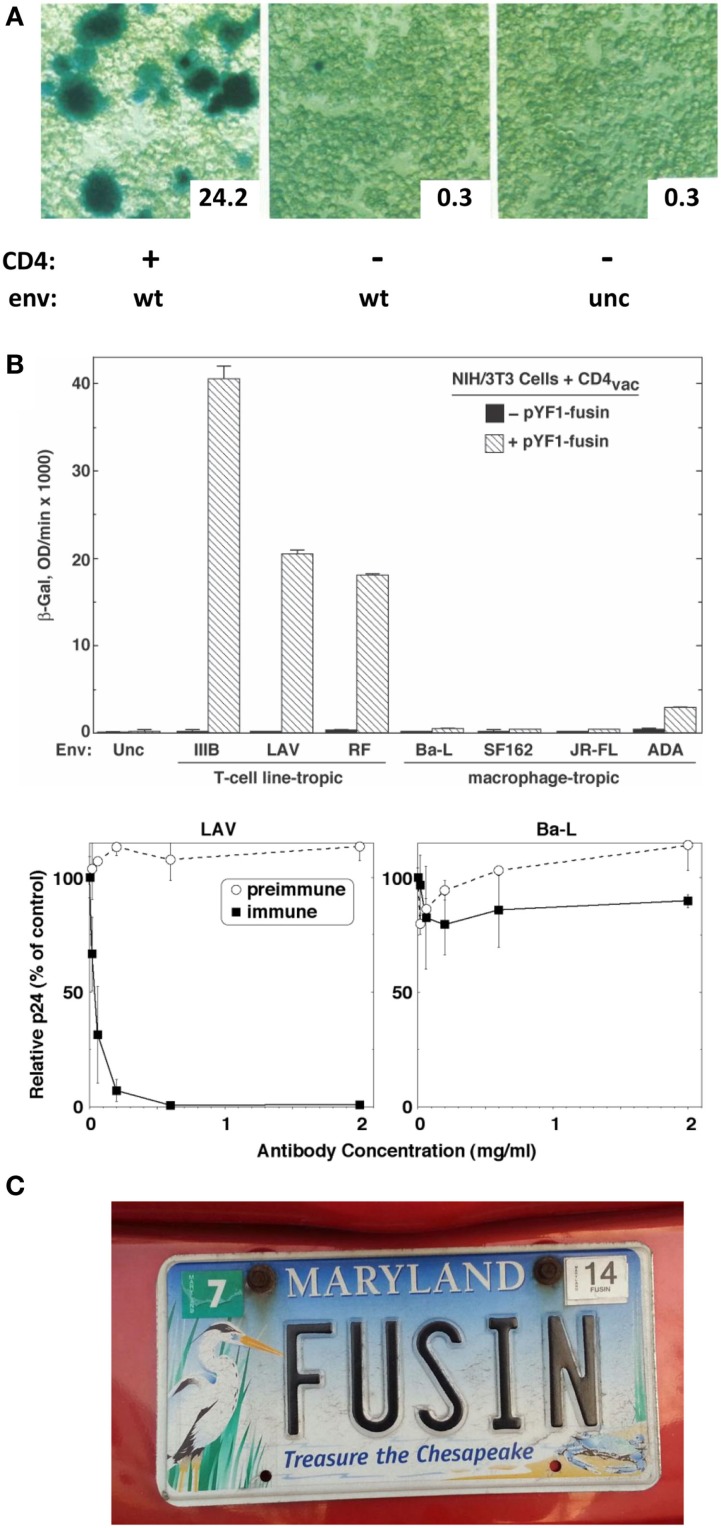
**(A)** Reporter gene assay for HIV-1 Env/CD4-mediated cell fusion. Effector HeLa cells expressed vaccinia-encoded HIV Env wild type (WT) or a non-functional uncleaved mutant (unc) and were transfected with a plasmid containing the *LacZ* gene linked to the T7 promoter. Target HeLa cells expressed vaccinia-encoded T7 RNA polymerase with (+) or without (−) CD4. Duplicate cell mixtures were incubated at 37°C for 2.5 h and β-galactosidase was measured in one set by *in situ* staining (photomicrographs) and in the other by colorimetric assay of detergent cell lysates (insets, arbitrary units). Adapted from Ref. (8). **(B)** Demonstration of fusin’s function as an entry receptor for TCL-tropic HIV-1. *Top panel*. Cell fusion assay. Effector NIH 3T3 cells expressed vaccinia-encoded Env from the indicated TCL-tropic or M-tropic HIV-1 isolate as well as T7 RNA polymerase. Target NIH 3T3 cells were co-transfected with the plasmid containing the *LacZ* gene linked to the T7 promoter plus either a control plasmid (filled bars) or a plasmid encoding fusin (cross-hatched bars). Cell mixtures were incubated at 37°C for 3 hr, and β-galactosidase was measured by the colorimetric assay of detergent cell lysates. *Bottom panel*. HIV-1 infection assay. PBMCs were pre-incubated with the indicated concentrations of purified rabbit antibodies [preimmune, and immune against the fusin N-terminus], then infected with HIV-1 LAV (left, TCL-tropic) or Ba-L (right, M-tropic). Culture supernatants were assayed by ELISA for p24 content at day 7. Results for each isolate are expressed as the percentage of p24 produced at each antibody concentration compared to the control value with no antibody. From Ref. (9). **(C)**. Rare detection of the name “fusin.” From the Maryland Department of Motor Vehicles.

The specificity of Env-mediated fusion/entry took on an additional layer of complexity beginning in the late 1980s with the growing awareness that different HIV-1 isolates displayed markedly distinct *in vitro* tropisms for infection of different CD4-positive target cell types (10). Some isolates infected CD4^+^ continuous T cell lines (and non-lymphoid human cell lines such as HeLa-CD4 transformants) but not primary macrophages; others displayed the reverse tropism, infecting primary macrophages but not CD4^+^ T cell lines. The terms “T cell line-tropic” (TCL-tropic) and “macrophage-tropic” (M-tropic) were used to distinguish these variants. Both phenotypes replicated in primary CD4^+^ T cells. This phenotypic distinction was more than simply a laboratory curiosity; in the real world of human HIV infection, the isolates obtained shortly after transmission and throughout the asymptomatic phase invariably displayed the M-tropic phenotype; TCL-tropic variants emerged only (years) later, during the transition to the symptomatic phase and progression to AIDS (and not in all cases). Studies from many groups in the early-mid 1990s pinpointed Env as the principle viral determinant mediating this tropism phenotype (4, 5). Using the reductionist cell fusion assay, Chris Broder in my group demonstrated a marked correlation between the fusion specificities of vaccinia-encoded HIV-1 Env glycoproteins and the infection tropisms of the strains from which they were derived (11). Subsequently, postdocs Ghalib Alkhatib and Chris performed fusion assays with transient hybrids between continuous cell lines and macrophages; the results suggested that the fusion specificities were attributable to distinct cellular cofactors (coreceptors?) mediating TCL- vs. M-tropism rather than to cell type-specific fusion restriction factors (12). Identification of these cofactors thus became the focal point of extensive searches by many groups worldwide; numerous candidate molecules were proposed (specific proteins, glycolipids), but these did not withstand detailed experimental scrutiny (4, 5).

Our initial identification efforts focused on the TCL-tropic cofactor, for the simple reason that it appeared to be expressed in diverse human cell lines (e.g., HeLa), thereby providing a technical advantage compared to primary macrophages. Yu Feng, a new postdoc in the group, initiated a strategy based on mRNA microinjection. At the outset, we committed to an unbiased approach with no preconceived notions about what type of protein we were seeking; our only criterion was gain-of-function in a fusion assay with CD4-expressing non-human host cells; microinjection of mRNA from a permissive human cell type (e.g., HeLa) should confer fusion-permissiveness. But what host cells to use? We knew that an NIAID investigator in a nearby lab, Phil Murphy, was doing microinjection experiments in *Xenopus* oocytes. Hearing that Phil was a highly congenial colleague, we approached him with the idea even though his research interests centered on a subject that had nothing to do with HIV, i.e., receptors for chemokines (small proteins that function as chemoattractants guiding leukocyte migration). Phil expressed enthusiasm, but we soon realized that the experimental features of the *Xenopus* oocyte system were incompatible with Env/CD4-mediated cell fusion. A more expeditious approach employing mammalian cells was required.

We then turned to the idea of transfecting a cDNA library from a fusion-permissive human cell type into a CD4-expressing non-human cell and testing for fusion gain-of-function. We knew that CD4-expressing HeLa cells were highly permissive fusion targets (presumably because of high cofactor expression) whereas CD4-expressing murine NIH 3T3 cells were consistently refractory (presumably cofactor-negative); moreover, a HeLa cDNA library was commercially available. We devised a functional screening assay involving transfection of the HeLa cDNA library into 3T3 target cells expressing vaccinia-encoded human CD4 (and T7 RNA polymerase); a small fraction of these cells would become fusion-permissive due to expression from the rare cDNA encoding the cofactor, and would fuse with added effector cells expressing a vaccinia-encoded TCL-tropic Env (and containing a transfected plasmid with the T7 promoter/*Lac Z* reporter). Staining *in situ* for β-galactosidase would reveal cell fusion. In the very first experiments (May 1995), the library-transfected target cells yielded decisively more β-galactosidase-positive cells compared to controls. After several rounds of library sub-fractionation and screening, a single cDNA clone was isolated that conferred robust fusion-permissiveness to the CD4-expressing murine cells.

DNA sequencing results obtained at the end of July 1995 indicated that the ~1.7 kb cDNA insert encoded a 352 amino acid protein with 7 putative transmembrane domains, i.e., a likely member of the G protein-coupled receptor superfamily. The nucleotide sequence had been reported by several groups during the previous 2–3 years, but the normal function of the protein was unknown. Since the only observed activity was in rendering CD4-expressing non-human cells permissive for HIV-1 fusion, we gave it the name “fusin.” During the following months, we accumulated critical experimental evidence proving fusin’s role as the sought-after entry cofactor for TCL-tropic HIV, including (a) gain-of-function experiments showing that fusin rendered CD4-expressing non-human cells permissive for HIV-1 Env-mediated cell fusion and virus infection, (b) specificity assays demonstrating fusion gain for TCL-tropic but not M-tropic Envs (Figure 1B, top), (c) loss-of-function experiments demonstrating the fusion-blocking and infection-neutralizing activity of rabbit antibodies against the putative N-terminal domain of fusin, and specificity based on selective antibody blocking for TCL-tropic but not M-tropic HIV-1 (Figure 1B, bottom), and (d) Northern blots demonstrating the presence of fusin mRNA in permissive human target cells and its absence from unusual non-permissive human targets (and, of course, from non-human cells). Taken together, these results convincingly established fusin as the critical entry cofactor for TCL-tropic HIV-1.

Some intriguing implications became apparent during the course of our work. First, the previous cDNA cloning papers indicated that the closest amino acid sequence homology with a protein of known function was with the human receptor(s) for interleukin 8, a CXC chemokine. How ironic, since one of the two back-to-back 1991 papers describing that first cloning of a human chemokine receptor was from none other than our nigh-collaborator Phil Murphy! Second, the possibility that fusin might be a chemokine receptor took on greatly added significance with a December 1995 paper from Paolo Lusso and Fiorenza Cocchi in Bob Gallo’s lab at the NCI, NIH; these investigators demonstrated that three CC chemokines, RANTES, MIP-1α, and MIP1-β accounted for the HIV-1 soluble suppressive activity released by CD8 T cells (13), a phenomenon first described by Jay Levy’s group during the preceding decade. Most interestingly, these CC chemokines suppressed a M-tropic much more than a TCL-tropic strain. Thus, the fusin discovery, together with the Lusso suppressive chemokines, provided a possible clue to the identity of the M-tropic cofactor: perhaps it was a chemokine receptor, in this case for RANTES, MIP-1α and MIP1-β.

I presented our fusin findings at a Keystone meeting in Santa Fe NM in February 1996, well before we were ready to submit the manuscript. Perhaps naively, I disclosed not only the evidence supporting fusin as the TCL entry cofactor but also the full amino acid sequence of the protein. The brush fire was now ignited, in both the HIV and chemokine research communities. But just in time for my group came the next irony. In late January 1996, we attended a seminar by Phil in which he revealed his lab’s cloning of a new chemokine receptor called CCR5, with precisely the specificity for the Lusso chemokines. Surely, there must be some connection with HIV, but what could that be? There we sat, with our knowledge of fusin, and our fledgling struggles to find the M-tropic cofactor by a similar functional cloning strategy using a cDNA library from primary macrophages. After some urgent pleas from the postdocs, I relinquished my stubborn adherence to the intellectual purity of the unbiased library screening approach and agreed instead to go for the direct kill. I contacted Phil in early March 1996, at last beginning a most productive collaboration. While attending another Keystone meeting at Hilton Head SC later that month, I phoned the lab and got the great news from Ghalib – he had the first data indicating a role for CCR5 as the M-tropic entry cofactor. The definitive experiments were completed over the next couple of months.

By the time, our fusin paper came out in May 1996 (9), the firestorms were raging in full. I give here only brief summaries, since there are fascinating stories to be told by other investigators who made major contributions to these developments [see reviews in Ref. (4, 5, 14, 15)]. On the HIV front, five independent papers (including ours) describing CCR5 as the essential entry cofactor for M-tropic HIV-1 were published within a week in June 1996. August–September 1996 saw the discovery of the CCR5 delta32 mutation, encoding a truncated non-functional protein; because of the high prevalence of this allele in Caucasian populations coupled with its simple Mendelian inheritance, CCR5 delta32 homozygosity provided the first and only molecularly understood mechanism for resistance to HIV infection. Moreover, this genotype was the basis for the first, and still only, documented cure of HIV infection. By October–November of 1996, both fusin and CCR5 were upgraded from cofactors to true “coreceptors,” based on demonstrations of their physical interactions with Env. The findings that coreceptor engagement occurs only after CD4 binding means that designation of CD4 as the primary receptor refers not only to its chronology of discovery but also to its obligate mechanism of action. In the ensuing nearly two decades, the coreceptor discoveries have engendered entirely new paradigms for understanding HIV transmission and pathogenesis, and have provided novel targets for antiretroviral drug development and gene therapy strategies aimed at curing HIV. In the chemokine field, our fusin paper was quickly followed (August 1996) by two back-to-back papers identifying the CXC chemokine stromal cell-derived factor 1 (SDF-1) as the natural ligand for fusin; SDF-1 was shown to inhibit TCL-tropic but not M-tropic HIV-1. Fusin was immediately renamed CXCR4 in keeping with chemokine receptor nomenclature. Thus, the impact of finding fusin/CXCR4, the first “2nd receptor” for HIV entry, endures to this day and likely well into the future. The fusin name, however, persists only in rare places (Figure 1C).

## Conflict of Interest Statement

The author declares that the research was conducted in the absence of any commercial or financial relationships that could be construed as a potential conflict of interest.
